# The Gaze of Schizophrenia Patients Captured by Bottom-up Saliency

**DOI:** 10.1038/s41537-024-00438-4

**Published:** 2024-02-20

**Authors:** Petr Adámek, Dominika Grygarová, Lucia Jajcay, Eduard Bakštein, Petra Fürstová, Veronika Juríčková, Juraj Jonáš, Veronika Langová, Iryna Neskoroďana, Ladislav Kesner, Jiří Horáček

**Affiliations:** 1 Center for Advanced Studies of Brain and Consciousness, National Institute of Mental Health, Klecany, Czech Republic; 2 Third Faculty of Medicine, Charles University, Prague, Czech Republic; 3 Institute of Computer Science of the Czech Academy of Sciences, Prague, Czech Republic; 4 Faculty of Electrical Engineering, Czech Technical University in Prague, Prague, Czech Republic; 5 Early Episodes of SMI Research Center, National Institute of Mental Health, Klecany, Czech Republic; 6 Department of Cybernetics, Faculty of Electrical Engineering, Czech Technical University, Prague, Czech Republic; 7 First Faculty of Medicine, Charles University, Prague, Czech Republic; 8 Faculty of Humanities, Charles University, Prague, Czech Republic; 9 Department of Art History, Masaryk University, Brno, Czech Republic

**Keywords:** Schizophrenia, Neuroscience, Psychology

## Abstract

Schizophrenia (SCHZ) notably impacts various human perceptual modalities, including vision. Prior research has identified marked abnormalities in perceptual organization in SCHZ, predominantly attributed to deficits in bottom-up processing. Our study introduces a novel paradigm to differentiate the roles of top-down and bottom-up processes in visual perception in SCHZ. We analysed eye-tracking fixation ground truth maps from 28 SCHZ patients and 25 healthy controls (HC), comparing these with two mathematical models of visual saliency: one bottom-up, based on the physical attributes of images, and the other top-down, incorporating machine learning. While the bottom-up (GBVS) model revealed no significant overall differences between groups (beta = 0.01, *p* = 0.281, with a marginal increase in SCHZ patients), it did show enhanced performance by SCHZ patients with highly salient images. Conversely, the top-down (EML-Net) model indicated no general group difference (beta = −0.03, *p* = 0.206, lower in SCHZ patients) but highlighted significantly reduced performance in SCHZ patients for images depicting social interactions (beta = −0.06, *p* < 0.001). Over time, the disparity between the groups diminished for both models. The previously reported bottom-up bias in SCHZ patients was apparent only during the initial stages of visual exploration and corresponded with progressively shorter fixation durations in this group. Our research proposes an innovative approach to understanding early visual information processing in SCHZ patients, shedding light on the interplay between bottom-up perception and top-down cognition.

## Introduction

Schizophrenia (SCHZ) is typically associated with deficits in domains related to information processing, such as perception, attention, working memory, and learning^1^. All these domains likely have one common denominator: impaired salience, the property by which something stands out from surrounding context. Salience is typically regarded as having two components: physical and cognitive salience. Physical salience refers to the aspects of a stimulus that automatically capture attention or direct gaze in a stimulus-driven, goal-independent, or bottom-up manner^2^. In contrast, cognitive salience is task-oriented, influenced by tasks assigned by external sources or driven by one’s current internal goals^3^. Disruption of physical salience, which is based on sensory sensitivity to external stimuli, may impede the formation of cognitive salience-related associations. This means that it can affect our ability to attribute meaning to individual stimuli from the external environment^4^. Kapur proposed that dysregulated, hyperdopaminergic states at the cellular level may lead to the attribution of aberrant salience to individual experiences at the psychological experiential level^5^. However, salience formation is a complex, long-term process that reflects our internal model of the world, which may not be stable in SCHZ due to distortions and instability of sensory signals^6^.

Vision is our most developed sense^7,8^ and unsurprisingly a substantial amount of brain processing is devoted to it, with over half the primate brain being involved in vision-related processing^9^. Due to the limited computational capacity of the visual cortex^10^, it is critical to correctly cluster visual percepts according to a hierarchy of importance. The internal model of the world is derived from the combination of neural filters and cognitive signals that gradually calibrate them. This mechanism allows the brain to process visual signals efficiently and to focus its limited computational capacity and attention only on those parts of the scene that are subconsciously assessed as important^11,12^. Computational capacity limits are mainly related to the physiological aspects of the neurons themselves and the functional circuits sensitive to the different elements of the visual scene^13,14^. The brain solves this limited capacity for attention allocation through prediction mechanisms^15^. The perceptual onset is preceded by a quick subliminal observation of the scene (bottom-up), which is based on its physical saliency (contrast, brightness, and low spatial frequencies). This observation helps us quickly orient ourselves and focus our attention in the next step, in which higher (top-down) cognitive processes come into play. These processes are related to the cognitive saliency formed by our internal model of the world^6,16^. Low spatial frequency (LSF) information is swiftly extracted from visual stimuli and conveys general details about the shape and orientation of objects within a scene. This LSF information subsequently contributes to the formation of top-down predictions, influencing visual attention and higher-level cognitive processes related to visual perception^16–19^. A primary outcome resulting from the disruption of this process is a disorder of attentional capacity and the inability to rapidly incorporate salient percepts into the stream of consciousness^20,21^.

In SCHZ, previous findings indicated a disruption in both types of processing: basal visual perception based on incorrect processing of visual stimuli (bottom-up)^22–25^, and impairment of higher visual cognition based on the processing of visual stimuli influenced and orchestrated by previous experience (top-down/feedforward sweep)^26–34^. The stimuli used in these experiments are typically designed based on the research question being addressed. Bottom-up experiments predominantly work with elementary stimuli, such as basic line figures^35^, Gabor patterns^29,36^, and pop-out structures^37^, while top-down experiments use different types of visual illusions^33,38^ or faces^39^. However, this approach falls short in providing a comprehensive mapping of the interplay between bottom-up and top-down processes during complex visual processing in everyday environments. It also lacks the capability to conclusively ascertain how deficits in bottom-up processing influence the perception, cognition and formation of aberrant saliency of complex real-life scenes in SCHZ population.

To address this knowledge gap, we attempt to identify differences between both groups by using recent saliency “bottom-up” and “top-down” predictive models^40,41^, with the former relying solely on physical visual properties and the latter additionally incorporating object recognition. Attention allocation has been intensively investigated through saliency models using “saliency maps”^42–44^, a computational concept that predicts graded saliency for each location of an image based on its low-level visual features, and thus predicts bottom-up attention^45^. It includes three components: (1) feature maps that represent fundamental visual characteristics such as color, orientation, luminance, and motion; (2) saliency maps resulting from combining normalized feature maps that highlight the visually significant areas in an image, solely based on their physical attributes, without taking into account any semantic features of the stimulus; (3) the “ground truth maps” representing the saliency maps derived from the real eye-tracking data capturing viewer attention allocation to specific regions of the image. The efficacy of saliency model predictions is then evaluated through its comparison with ground truth maps. In previous studies, saliency models have even been employed to analyze brain activity in response to visual stimuli, with distinct brain areas linked to the ‘saliency map’ generated by a saliency model^46,47^.

Recent technological advances in the field of machine learning have enabled the incorporation of additional convolutional neural network (CNN) layers to original bottom-up models. These added CNN layers reflect top-down cognition, which is involved in analysis and categorization of specific semantic content of a scene (e.g., objects, faces, emotions)^48–51^. However, it is important to emphasize that such models are not solely based on top-down cognition; they still incorporate the bottom-up layer within their computations. In this paper, for the sake of simplicity, we refer to such models as “top-down” because, unlike bottom-up models, they have the capability to suppress the bottom-up component in favour of top-down processing^52,53^.

We utilized these two models to determine the likelihood of an observer directing their attention to specific areas within the scene. We expect that analyzing ground truth maps derived from eye-tracking data of individuals with schizophrenia (SCHZ) and healthy controls (HCs), and comparing these with mathematically predicted saliency, will provide deeper insights into the similarities and differences in bottom-up and top-down visual processing between these two groups. We hypothesized that SCHZ patients’ attention is influenced more by the physical properties of the image than HC’s attention. This suggests a tendency to prioritize highly physically salient percepts in the scene more than HC^54–57^, likely reflecting the disruption of higher cortical processes consistently found across studies and resulting in the expected lower predictive ability of the top-down model in SCHZ patients^58–60^. In this paper, we employ the term “bottom-up bias” to denote a tendency to prioritize bottom-up signal over top-down processing^61^.

To investigate the ‘bottom-up bias’ in schizophrenia (SCHZ), our approach involved a multi-faceted comparison using saliency models across both SCHZ patients and HCs. Initially, we compared the overall results of these models between the two groups. Furthermore, our analysis extended to assessing the performance of the saliency models across five specific content-based categories, each inherently linked to either bottom-up or top-down processing. This nuanced categorization allowed us to parse the visual processing mechanisms more precisely and understand how each model interprets different types of visual stimuli in SCHZ and HCs. Subsequently, we integrated a stepwise analysis of two consecutive time periods in our study – the first encompassing up to five fixations, and the second starting from the sixth fixation. This sequential analysis was aimed to unravel the dynamics of visual perception in SCHZ. By examining these two distinct phases, we sought to identify and contrast the engagement of bottom-up and top-down components in the visual perception processing of both groups. Finally, to reveal confounding factors that might influence the results of the two saliency models, we decided to test the relationship of oculomotor movements with psychological metrics (Continuous Performance Test (CPT) and Positive and Negative Syndrome Scale (PANSS)), medication, disease duration, and the length of its untreated phase (DUP).

## Results

### Differences in the Performance of Saliency Models

Comparison of saliency maps calculated for each participant (ground truth maps) to saliency predictions lead to 13,436 normalized scan path (NSS) values from 53 subjects (28 SCHZ, 25 HC). A direct nonstatistical comparison of the NSS scores between two saliency models showed that the bottom-up (GBVS) model was able to predict oculomotor behavior better in the SCHZ population (M = 1.43, SD = 0.58) than in HC (M = 1.35, SD = 0.51). In contrast, the top-down (EML-Net) model better predicted the distribution of fixations in HC (HC: M = 2.16, SD = 1.13) than SCHZ (SCHZ: M = 2.08, SD = 1.29). However, when we employed linear mixed effects models (LME) for statistical comparison, the analysis did not corroborate the differences observed in the direct, non-statistical comparison of NSS scores between groups and across models.

Evaluation of NSS scores for the bottom-up (GBVS) model did not show significant differences between-groups but indicated significantly higher performance of SCHZ patients in the highly salient image category (Table 1). The top-down (EML-Net) model also did not show an overall between-groups effect but showed significantly lower patients’ performance in images depicting social interactions (Table 1).

**Table 1:** Results of LME comparison for top-down and bottom-up model.

	bottom-up sqrt(NSS)			top-down sqrt(NSS)		
*Predictors*	*Estimates*	*CI*	*p*	*Estimates*	*CI*	*p*
(Intercept)	0.44	0.37–0.58	<0.001	0.57	0.53–0.61	<0.001
SCHZ	0.01	−0.01–0.03	0.281	–0.03	−0.07–0.02	0.206
Incongruent	0.01	−0.10–0.11	0.921	0.04	−0.01–0.09	0.132
Physically salient	−0.04	−0.14–0.06	0.428	–0.11	−0.17–−0.06	<0.001
Social interaction	−0.08	−0.18–0.02	0.099	0.18	0.12–0.23	<0.001
Social landscape	−0.02	−0.12–0.08	0.699	0.09	0.04–0.14	0.001
SCHZ × Incongruent	0.01	−0.01–0.02	0.224	0.03	−0.00–0.06	0.050
SCHZ × Physically salient	0.02	0.00–0.03	0.015	0.03	−0.00–0.06	0.051
SCHZ × Social interaction	0.01	−0.01–0.02	0.324	–0.06	−0.09–−0.03	<0.001
SCHZ × Social landscape	0.01	−0.00–0.03	0.153	0.01	−0.02–0.04	0.582
**Random Effects**						
σ^2^	0.02			0.07		
τ_00_	0.00 _ID_			0.01 _ID_		
	0.00 _imageCat_			0.00 _imageCat_		
ICC	0.10			0.07		
N	54 _ID_			54 _ID_		
	5 _imageCat_			5 _imageCat_		
Observations	13436			13436		
Marginal R^2^/Conditional R^2^	0.049/0.140			0.090/0.157		

*sqrt* square root, *NSS* normalised scan path, *ID* unique participant identification string, *imageCat* Image category.

At the whole-group level, including both SCHZ and HC, the bottom-up (GBVS) model showed no differences between image categories. On the other hand, the top-down (EML-Net) model showed lower prediction capability in the physically salient image category, and higher capability in the social interaction and social landscape image categories (Table 1).

### Between-group differences in bottom-up and top-down predictions in time

**Fig. 1:**
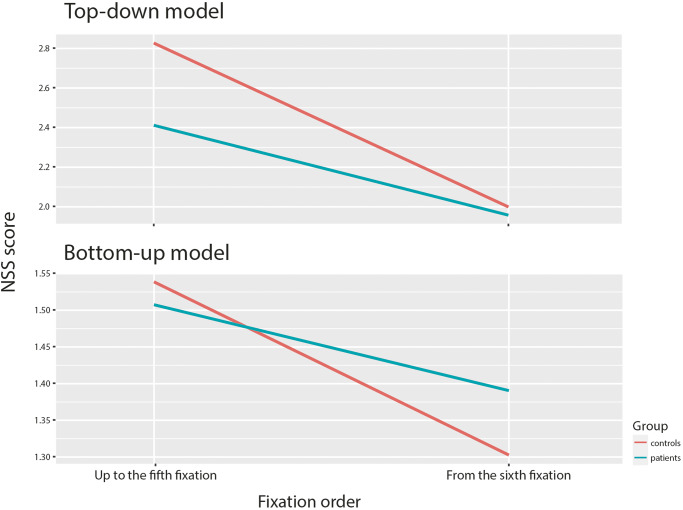
The difference between models performance in time. A difference in NSS score of the top-down and bottom-up model between-groups over time. **Description:** The top-down (EML-Net) model performs better within both time periods in the case of HCs. The bottom-up model, on the other hand, is better in predicting saliency in the SCHZ population only in the case of the second period from the sixth fixation. In the first period, the prediction is more accurate for HCs than SCHZ patients.

**Fig. 2:**
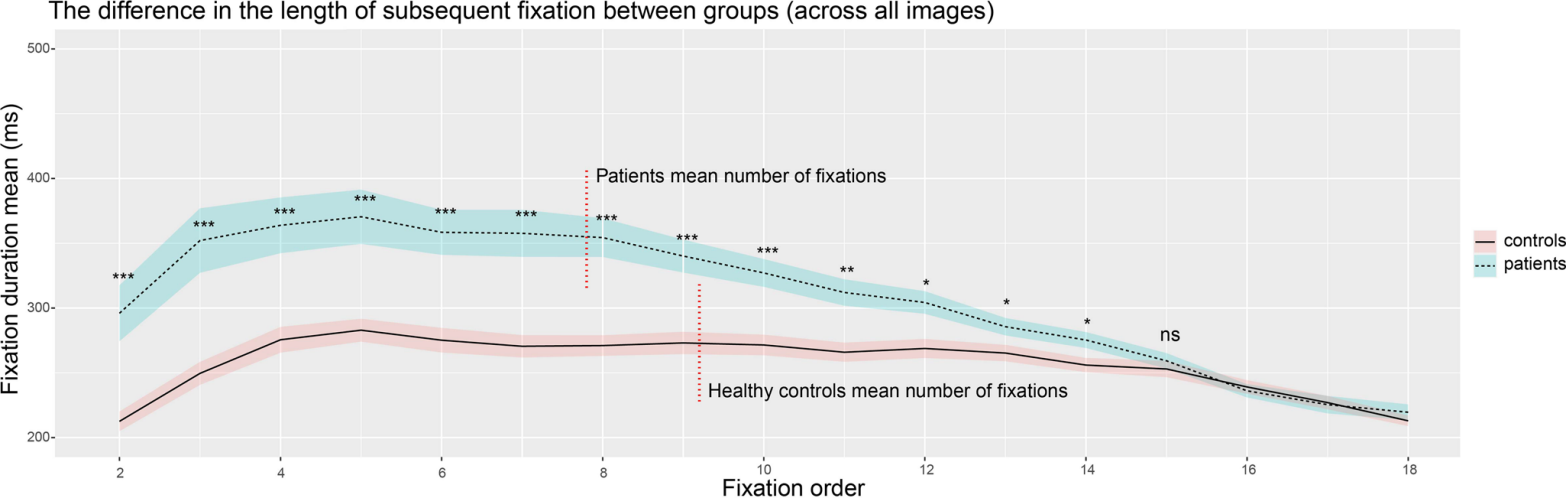
Inter-group differences in the duration of individual fixations (group mean, standard error of the mean). Vertical red dotted lines show the mean number of fixations in groups ***p < 0.001; **p < 0.01; *p < 0.05; *ns* = not significant. A sequential testing procedure was applied to control false positive rate – stopping at the first fixation with a non-significant result.

To identify the inter-group differences in the involvement of bottom-up and top-down processes over time, we calculated NSS score for each model in two different time periods: up to the fifth fixation and from the sixth fixation (Fig. 1). The decision to split the dataset into two periods was based on previous research showing that prediction accuracy for bottom-up models is lost around the fifth fixation^62^. Another decision that led us to split the dataset is the peak of the fixation duration, which is located just around the fifth fixation, for both groups (Fig. 2). We applied LMER models to both periods and both saliency models.

#### Sequential analysis of bottom-up (GBVS) model

The LME model revealed no significant differences in NSS scores between the SCHZ and HC groups for either observed period. However, in the context of physically salient images, the model consistently showed a better prediction of oculomotor behavior for SCHZ patients compared to HCs, in both periods (Table 2).

**Table 2:** Differences in NSS scores between SCHZ and HC groups, for bottom-up (GBVS) model in two different time periods.

	bottom-up sqrt(NSS) – To the fifth fixation			bottom-up sqrt(NSS) – Up to sixth fixation		
*Predictors*	*Estimates*	*CI*	*p*	*Estimates*	*CI*	*p*
(Intercept)	1.61	1.51–1.70	<0.001	1.54	1.52–1.57	<0.001
SCHZ	−0.02	−0.04–0.00	0.093	0.01	−0.0–0.04	0.270
Incongruent	0.03	−0.10–0.16	0.622	−0.00	−0.03–0.03	0.953
Physically salient	−0.06	−0.19–0.07	0.393	0.04	−0.07–-0.02	0.002
Social interaction	−0.10	−0.23–0.03	0.129	−0.10	−0.13–−0.07	<0.001
Social landscape	−0.01	−0.14–0.12	0.925	−0.03	−0.06–−0.00	0.039
SCHZ × Incongruent	−0.01	−0.03–0.02	0.595	0.01	−0.01–0.03	0.193
SCHZ × Physically salient	0.02	0.00–0.05	0.030	0.02	0.00–0.04	0.046
SCHZ × Social interaction	0.01	−0.01–0.03	0.310	0.01	−0.01–0.03	0.306
SCHZ × Social landscape	0.01	−0.02–0.03	0.532	0.02	−0.00–0.04	0.107
**Random Effects**						
σ^2^	0.04			0.03		
τ_00_	0.00 _ID_			0.00 _ID_		
	0.00 _imageCat_			0.00 _imageCat_		
ICC	0.06			0.05		
N	54 _ID_			54 _ID_		
	5 _imageCat_			5 _imageCat_		
Observations	13435			13097		
Marginal R^2^/Conditional R^2^	0.040/0.097			0.039/0.087		

*sqrt* square root, *NSS* normalised scan path, *ID* unique participant identification string, *imageCat* Image category.

Furthermore, an analysis of the second period revealed differential performance across image categories at the whole-group level. Specifically, the bottom-up model indicated better performance for physically salient images, while it showed reduced effectiveness in accurately predicting oculomotor movements for stimuli depicting social interactions and social landscapes (Table 2).

#### Sequential analysis of top-down (EML-Net) model

LME results showed a difference in NSS score between groups during the first time period (Table 3). We also observed significantly higher model predictive performance of patients’ oculomotor behavior in the physically salient image category and lower performance in social landscape images category in the first period. Stimuli depicting social interactions had significantly lower NSS score in SCHZ patients in both periods (Table 3). Contrastingly, when we examined the whole-group level results, which include both SCHZ and HC groups, no differences were observed between image categories in either of the two periods (Table 3).

**Table 3:** Differences in NSS scores between SCHZ a HC groups for top-down (EML-Net) model in two different time periods.

	top-down sqrt(NSS) – To the fifth fixation			top-down sqrt(NSS) – Up to sixth fixation		
*Predictors*	*Estimates*	*CI*	*p*	*Estimates*	*CI*	*p*
(Intercept)	1.81	1.35–2.27	<0.001	1.67	1.36–1.98	<0.001
SCHZ	−0.11	−0.17–−0.04	0.001	−0.02	−0.08–0.03	0.431
Incongruent	0.14	−0.51–0.79	0.679	0.02	−0.42–0.46	0.936
Physically salient	−0.14	−0.79–0.51	0.663	−0.13	−0.57–0.31	0.557
Social interaction	0.25	−0.40–0.90	0.443	0.20	−0.24–0.64	0.370
Social landscape	0.25	−0.40–0.89	0.460	0.05	−0.39–0.49	0.826
SCHZ × Incongruent	−0.01	−0.05–0.04	0.706	0.04	0.00–0.08	0.034
SCHZ × Physically salient	0.05	0.00 – 0.09	0.029	0.02	−0.01–0.06	0.232
SCHZ × Social interaction	−0.04	−0.09–−0.00	0.045	−0.08	−0.12–−0.05	<0.001
SCHZ × Social landscape	−0.05	−0.09–-0.00	0.035	0.01	−0.02–0.05	0.521
**Random Effects**						
σ^2^	0.16			0.12		
τ_00_	0.01 _ID_			0.01 _ID_		
	0.05 _imageCat_			0.02 _imageCat_		
ICC	0.28			0.22		
N	54 _ID_			54 _ID_		
	5 _imageCat_			5 _imageCat_		
Observations	13435			13097		
Marginal R^2^/Conditional R^2^	0.086/0.346			0.054/0.263		

*sqrt* square root, *NSS* normalised scan path, *ID* unique participant identification string, *imageCat* Image category.

### Group Differences in Fixation and Explored Area of the Image

The SCHZ group showed a significantly lower mean number of fixations per image than the HC (SCHZ: M = 8.92, SD = 1.28; HC: M = 9.22, SD = 0.75; *t*(54) = 5.26, *p* < 0.001), and the overall mean fixation duration was longer in SCHZ than in HC (SCHZ: M = 326.12 ms, SD = 22.97; HC: M = 254.83 ms, SD = 24.15; *t*(54)= −4.44, *p* < 0.001). We also observed a statistically significant difference between the groups in terms of the total area of the image that received fixations. This ‘total fixed image area’ refers to the cumulative portion of the image that was the focus of gaze fixations across all participants within each group. The standard deviation (SD) test revealed that the SCHZ group had significantly reduced spread of fixations over the image area (SCHZ: SD Mean = 678.28; SD = 76.3; HC: SD Mean = 727.56 (SD = 83.82); t(54) = 6.87, *p* < 0.001).

In addition, we identified between-group differences in the temporal dynamics of fixation duration. In SCHZ, the average fixation duration stabilized after an initial increase in duration. Around the fifteenth fixation, their duration became comparable to HC. The fifth fixation was achieved in 99% of all trials in HC and in 96% of all trials in SCHZ. Tenth fixation was achieved in 96% of all trials in HC and in 82% of all trials in SCHZ. Fifteenth fixation was achieved in 79% of all trials in HC and in 45% of all trials in SCHZ. A sequential testing procedure was used to test the significance of this difference. The first fourteen fixations showed a statistically significant difference in fixation lengths (*t*(54) = −2.55, *p* = 0.013). The fifteenth and subsequent fixation durations did not differ between groups (*t*(54) = −1.67, *p* = 0.098) (Fig. 2).

In the SCHZ group, we also investigated the relationship between oculomotor movements (including the duration and number of fixations) and various factors: the antipsychotic medication dosage, responses on the PANSS questionnaire, the duration of illness, and the period of untreated illness. However, our analysis revealed no statistically significant correlations between these variables and oculomotor movements. Additionally, we examined the relationship between oculomotor movements and CPT test results in both SCHZ and HC groups. We found a negative correlation between CPT Commissions and the mean number of fixations in HC group, but no other significant correlations with other measured variables and participant groups. Detailed results can be found in (Table 4).

**Table 4:** Results of psychological measurements.

Group	SCHZ				HC			
Variable	Mean of fixation number		Mean of fixation		Mean of fixation number		Mean of fixation duration	
	Pearson Correlation r(28)	*p*-value	Pearson Correlation r(28)	*p*-value	Pearson Correlation r(23)	*p*-value	Pearson Correlation r(23)	*p*-value
CPT omissions	0.12	0.52	−0.08	0.88	−0.19	0.34	0.24	0.23
CPT commissions	0.15	0.45	−0.16	0.4	−0.51	0.01	0.36	0.07
CPT hit reaction time (HRT)	−0.18	0.36	0.27	16	0.17	0.4	−0.13	0.54
CPT HRT standard deviation	−0.2	0.29	0.21	0.26	−0.26	0.21	0.05	0.86
CPT variability	−0.22	0.26	0.22	0.21	−0.24	0.23	0.13	0.53
CPT detectability	0.13	0.5	−0.09	0.64	−0.35	0.08	0.31	0.12
CPT perseverations	0.19	0.32	−0.21	0.26	0.28	0.17	−0.2	0.33
CPT HRT block change	−0.13	0.52	0.22	0.24	−0.05	0.8	−0.15	0.47
CPT HRT inter-stimulus	−0.19	0.33	0.15	0.44	−0.05	0.8	0.05	0.67
PANSS positive symptoms	−0.04	0.84	0.01	0.96	NA	NA	NA	NA
PANSS negative symptoms	−0.17	0.37	0.09	0.64	NA	NA	NA	NA
PANSS general psychopathology	−0.14	0.48	0.07	0.72	NA	NA	NA	NA
PANSS total score	−0.17	0.34	0.11	0.59	NA	NA	NA	NA
Duration of illness (months)	−0.08	0.64	0.17	0.36	NA	NA	NA	NA
Duration of untreated psychosis (months)	−0.11	0.54	0.2	0.28	NA	NA	NA	NA
CHLPMZ equivalent	−0.2	0.29	0.31	0.9	NA	NA	NA	NA

*CPT* Conners’ Continuous Performance Test III, *PANSS* Positive and Negative Syndrome Scale, *NA* notavailable.

## Discussion

The main finding of our study is that the bottom-up model was able to better predict the oculomotor behavior of the SCHZ population and in contrast the top-down model better predicted the oculomotor behavior of HCs. While the LME model did not statistically confirm differences for either the bottom-up or top-down models overall, it identified significant variations upon examining specific image categories. These findings indicate that the bottom-up model better predicted oculomotor behavior in SCHZ patients compared to HC when viewing physically salient images. This observation supports a ‘bottom-up‘ bias in SCHZ patients and the assumption of a delayed integration of visual signals initially processed by bottom-up mechanisms into the subsequent top-down processing^26,55,56^.

On the other hand, the top-down model was more effective in predicting the gaze patterns of SCHZ patients compared to HCs when they viewed incongruent scenes. This observation suggests that although the model is capable of predicting gaze patterns in relation to the objects within a scene, it falls short in recognizing the incongruity of these objects, that is, an understanding how the objects relate contextually. This observed behavior is likely because the top-down model, which inherently lacks the ability to assess the semantic context of objects, does not factor in the presence of incongruent objects within its predictive framework. In essence, the model’s limited capacity to evaluate semantic contexts aligns with the similar cognitive limitation observed in SCHZ patients^63^. Therefore, the enhanced predictive accuracy of the top-down model for SCHZ patients may stem from this shared deficiency in correctly interpreting the semantic context of objects, resulting in more accurate oculomotor predictions for this group. Our findings also indicate that the top-down model more accurately predicted the oculomotor behavior of HCs compared to SCHZ patients in the context of social interactions images. This is consistent with earlier research highlighting the impaired ability of SCHZ patients to process more complex visual scenes such as social interactions and emotions^64–66^. This outcome is linked to negative symptoms of emotional blunting^67^ and a deficit in processing the low spatial frequency (LSF) of images^68,69^.

Category-specific stimuli analyses showed better performance in SCHZ group for the top-down model in categories of social interaction and social landscape. This finding is in agreement with previous reports on the properties of saliency models^70,71^. This enhanced prediction accuracy suggests that this model excels in accounting for higher cognitive processes associated with the interpretation of individuals and objects within the scene and their interactions. Conversely, the performance of the top-down model was less effective in predicting the oculomotor behavior of HCs in response to physically salient stimuli. The top-down model’s reduced capacity to predict oculomotor behavior for physically salient stimuli reaffirms its overall lower sensitivity to the bottom-up component within the predicted saliency map.

As expected, the temporal analysis of the models allowed us to reveal how top-down and bottom-up processes are involved in cognition and its formation in the groups we studied. The bottom-up (GBVS) model indicated no significant differences between the groups across both periods. However, this trend changed when we focused on specific stimulus categories. Notably, for physically salient images, the GBVS model consistently showed better performance in SCHZ patients than in HCs during both periods. This confirms the previously reported tendency of SCHZ patients to focus their attention on physically salient stimuli^72,73^. The second analysis shows a difference in performance of the top-down (EML-Net) model between groups. Especially in the first period, the nuanced differences in how SCHZ and HC groups process visual information is highlighted. This distinction, particularly evident in the early period, underscores a potential divergence in cognitive processing strategies between the two groups. As the model’s ability to differentiate between SCHZ and HC partly diminishes in the second period, it suggests a partial convergence in visual processing strategies over time, or possibly an adaptation in the SCHZ group’s visual attention mechanisms. Differences persist for images depicting social interaction and emerge in incongruent images category.

Furthermore, these observations are in agreement with results from the CPT, where SCHZ patients exhibited higher rates of omission and perseveration errors compared to HCs. These CPT findings imply a greater tendency of SCHZ patients to overall inattentiveness (as indicated by higher omission scores) and to the use of more automatic responses (as evidenced by higher perseveration scores). Together, these elements suggest an impaired ability of SCHZ patients to direct their focus towards visual stimuli^74^. This impairment may also contribute to the delayed scene orientation observed in SCHZ patients, thereby affecting the efficiency of bottom-up signal processing. In the HC population, after the initiation phase, bottom-up saliency is suppressed by the top-down saliency of higher cognitive processes^16,75–77^, but as seen in the results it appears that this onset is delayed in the SCHZ population.

The delayed emergence of top-down cognitive processes is likely attributable to dysfunctions in LSF processing. LSF processing is essential for swift scene orientation, laying the groundwork for top-down predictive mechanisms and focused attention distribution within the visual scene^16^. The absence of notable differences between-groups in the second period of top-down model predictions implies that the slower initiation of top-down cognition might be linked to LSF processing abnormalities repeatedly reported in SCHZ population^61,78–80^. Previous studies mainly focus on the reduced ability of the SCHZ population to process LSFs, which has been attributed to dysfunction of the magnocellular optical pathways. However, recent findings indicate that LSFs may not be processed only by the magnocellular pathways but are likely processed in parallel in the koniocellular pathways^81,82^. Consequently, the research focus has shifted toward the retina itself in recent years^83–85^. One possible reason for the slower bottom-up signal processing in SCHZ is the inflammatory processes of retinal microvascularity, which are associated with commonly reported atrophy of retinal nerve fibers^86,87^. The outcome of this process is a low signal-to-noise ratio^88^, particularly resulting in an increased level of vagueness related to the nature of a percept/signal, ultimately leading to a disruption of the decision-making process^89^. However, inflammatory processes and associated atrophy would not explain why, in early-stage and untreated first-episode patients, hypersensitivity is often encountered^55,57^. Retinal atrophy can only explain the later stages of the illness when hypersensitivity eventually progresses to hyposensitivity, which also extends to other frequencies of the visual scene^55,90,91^. An alternative explanation that would also include hypersensitivity to LSFs would be instability in retinal dopamine levels^6^. Dopamine influences the size of receptive fields, thereby affecting the sensitivity to individual frequencies of the perceived image^92^. Increased dopamine levels reduce the size of receptive fields, leading to increased sensitivity to high spatial frequencies and vice versa^93,94^. Therefore, the instability of the receptive fields may contribute significantly to the formation of the aberrant salience that is typical for schizophrenia^6^.

In our study, the SCHZ patient group exhibited fewer yet longer fixations compared to the HC group, corroborating findings from existing literature^95–97^. While previous studies have suggested a link between these oculomotor differences and the severity of both negative and positive SCHZ symptoms, the nature of this association remains a subject of debate^98^. In contrast to these studies, our results did not establish a connection between the severity of SCHZ symptoms (whether negative or positive) and oculomotor behavior. This absence of correlation extended to the outcomes of the PNASS as well as to medication effects. Furthermore, we observed no significant relationship between fixation patterns and CPT performance within the SCHZ group. These findings imply that the overall ability of SCHZ patients to sustain attention does not significantly impact the results of predictive models. It raises the possibility that these specific differences in saliency and its predictive model might be considered as trait markers of SCHZ itself.

Temporal analysis of fixation duration revealed a diminishing difference between the HC and SCHZ groups over time. Initially, the SCHZ group exhibited prolonged fixations, likely indicative of extended time needed for scene orientation and LSF signal processing. However, fixation durations gradually decreased, suggesting the engagement of advanced top-down cognitive processes. This pattern aligns with the documented reduction in fixation duration and count in SCHZ during top-down cognitive tasks, such as object search or fixation within a scene^99^. This “unknown compensatory mechanism”, as the authors of the original study called it, might relate to altered receptive field sensitivity, potentially due to dopamine fluctuations in the retina and variations in retinal morphology, affecting receptive field distribution and size. However, a precise answer to this question would require more in-depth research.

In this study, we explored the application of salience models in schizophrenia (SCHZ) research, an area with limited prior investigation^100,101^. Our findings indicate that predictive models of visual saliency are potent tools for identifying errors in visual information processing and the development of aberrant saliency in SCHZ patients. Emphasis should be placed on incongruent stimuli, stimuli that are physically salient, and complex stimuli depicting social interactions. These types of stimuli effectively illustrate the limitations of the models and the specific abnormalities in visual processing among the SCHZ population. Our study also reveals that the previously documented bias in SCHZ patients towards bottom-up signals^31,55,57,61,102,103^ is variable over time, possibly originating from disruptions in early-stage visual processing. This disruption might further impede the onset of top-down visual cognition. The altered and prolonged processing of bottom-up signals likely leads to flawed and unstable internal representations of the world, impacting higher cognitive functions^6^. Our study highlights the complex interaction between bottom-up and top-down processes in the visual signal processing of SCHZ patients, marked by a progressive decrease in fixation duration. However, to fully comprehend these intricate dynamics, further research is essential.

### Limitations

The first limitation of the presented study arises from the above-mentioned question: to what extent the presented saliency models reflect purely “bottom-up” and “top-down” processing? Although this is still a matter of debate, the proportion of these two components largely differs in the applied models and thus the presented methodology can describe the differences between HC and SCHZ bottom-up and top-down processing. Also, the top-down EML-Net model, having been trained on data from individuals without neurological conditions, presents a challenge in interpretation: it’s unclear whether the improved model fit observed in the control group is due to differences in the type of top-down information prioritized by patients and controls, or if it simply reflects variances in the degree to which they prioritize such information. This ambiguity raises questions about the model’s ability to accurately capture the nuances of top-down information processing in populations with neurological conditions like SCHZ. Other limitation pertains to the antipsychotic treatment of SCHZ participants. The relationship between antipsychotic medication and oculomotor movement is a controversial topic which has been questioned before^104–106^, and our results support these concerns.

## Methods

### Participants

This study involved 62 subjects (37 SCHZ and 25 HC) (Table 5), matched in age, sex, and years of education (within ± 2 years). Some HCs were matched to a larger number of SCHZ patients due to the lower availability of HCs with fewer years of education, resulting in this imbalance. The number of participants was estimated by a power analysis (Appendix A). Nine participants (9 SCHZ, 0 HC) were excluded due to incorrect eye-tracking measurements (within the measurement, the calibration deviation increased to more than 0.5°; high blink rate; fatigue; and concentration problems). Participants were recruited into the study as part of the Early-Stage Schizophrenia Outcome (ESO) Study^107–109^ and through the National Institute of Mental Health clinic, Czech Republic (NIMH CZ). The diagnostic procedure was standardized with the structured Mini-International Neuropsychiatric Interview^110^, and patients were diagnosed according to ICD-10^111^. Only patients diagnosed with schizophrenia spectrum disorder were included in the analyses (i.e., F20, F23 and F25)^111^. Additional inclusion criteria were age between 18 and 60 years, the absence of severe neurological illness or organic brain problems, and normal color vision as determined by the Ishihara test^112^. All the patients took medication at the time of participation. HCs were recruited via an advertisement from a similar socio-demographic background to the SCHZ participants. HCs were not allowed to have a history of psychiatric disorders (evaluated with a modified version of the M.I.N.I.) or in their first- and second-degree family members (assessed by an anamnestic questionnaire). Both groups were recruited between 2018 and 2021. The ethics committee of the NIMH CZ approved the study. All the experiments were performed in accordance with the relevant guidelines and regulations. Written, informed consent was obtained from all the subjects after receiving a complete study description. Participation in the research was voluntary, with a financial compensation of 500 CZK. In the SCHZ group, the current clinical condition and medication dose were also taken into consideration.

**Table 5:** Demographic and clinical characteristics of the experimental groups.

Variable	SCHZ (*n* = 30)	HC (*n* = 25)	*p*-value
	Mean (SD)	Mean (SD)	
Gender (F/M)	10/20	10/15	0.817
Age (years)	32 (9.1)	31.57 (7.57)	0.837
Education (years)	14.11 (2.64)	14.28 (2.15)	0.777
PANSS total score	37.6 (7.43)		
PANSS positive symptoms	8.18 (1.1)		
PANSS negative symptoms	11.06 (4.7)		
PANSS general symptoms	18.53 (3.03)		
CPT omissions	55.43 (14.84)	47.15 (4.63)	0.017
CPT perseverations	54.84 (11.55)	48.61 (7.81)	0.015
CPT commissions	54.62 (9.72)	53.15 (10.99)	0.583
CHLPMZ equivalent	399.1 (182.14)		
Duration of untreated psychosis (months)	5.12 (8.03)		
Duration of illness (months)	133.72 (170.45)		
Ratio of individual SCHZ diagnoses	F20 (*n* = 20); F23 (*n* = 10); F25 (*n* = 0)		

*CHLPMZ* Chlorpromazine.

### Visual stimuli selection and pre-processing

**Fig. 3:**
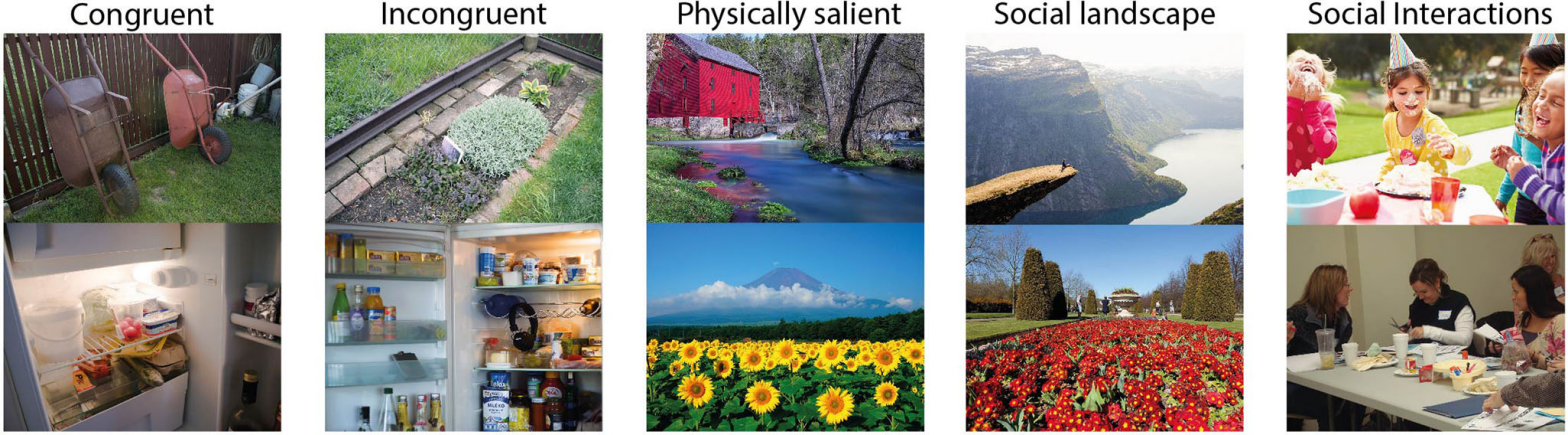
Examples of stimuli utilized in the experiment. The photographs were categorized into five different groups based on their content. (1) Everyday Scenes (Congruent) include images of typical, everyday environments where all elements are contextually appropriate and consistent. (2) Incongruent images contain everyday scenes but with objects that are contextually out of place or unusual. (3) Natural Scenes with Physically Salient Elements include natural environments that include elements with notable physical salience. (4) Scenes Depicting Social Interactions comprises scenes depicting social interactions. (5) Social Landscapes are natural scenes that include elements of nature, but feature also humans.

A total of 250 color images of an everyday naturalistic scene were used in the study. All the photographs were downloaded from public databases (Flicker, World Images, and Vecteezy) or taken by the study’s authors. The stimuli were divided into five categories (50 images pear each), based on their content (congruent, incongruent, physically salient, social landscape, social interaction) (Fig. 3). (1) Everyday Scenes (Congruent): This category includes images of typical, everyday environments where all elements are contextually appropriate and consistent. Such congruent scenes are expected to align well with top-down models’ predictions, as they match usual expectations of everyday environments. (2) Incongruent images: These scenes contain everyday settings but with objects that are contextually out of place or unusual. The incongruence of these objects is anticipated to challenge top-down models, which rely on contextual appropriateness, and could be more accurately predicted for individuals with SCHZ than HC due to the expected bottom-up bias in SCHZ^56^. (3) Natural Scenes with Physically Salient Elements: Scenes in this category are natural environments that include elements with notable physical salience—like unusual color, contrast, or orientation. These elements are expected to be more effectively predicted by bottom-up models, and thus potentially better predicted for individuals in the SCHZ group. (4) Scenes Depicting Social Interactions: This category comprises scenes focused on social interactions. These types of stimuli are expected to be more accurately predicted by top-down model for the HC group, as they involve understanding social cues and contexts. 5) Social Landscapes: These are natural scenes that include elements of nature and feature humans. Termed “social landscapes,” these scenes are anticipated to align better with top-down model predictions for the HC group, as they combine elements of nature with social interactions.

The Shine toolbox^113^ for MATLAB was used to normalize all the stimuli to color and luminance. Then two saliency models, Expandable Multi-Layer NETwork (EML-Net) and Graph-Based Visual Saliency Model (GBVS) (See below in section 4.6), were applied to each photograph, producing one saliency map per image and model. Subsequently, a black border was added to each image to reach a resolution of 3840 × 2160 pixels. The original mean image area was *M* = 6,029,277.12 pix, SD = 818,762.31. The mean area of the added black borders was *M* = 1,487,522.88 pix, SD = 818,762.31. The image area therefore occupied approximately 80% of the monitor area. The experiment was created and presented using SR Research Experiment Builder 2.3.1^114^ .

### Eye-tracking data acquisition

Eye movements were recorded using the EyeLink 1000 Plus eye tracker (SR Research Ltd. Ottawa, Ontario, Canada). The eye-tracker samples raw gaze data at 1000 Hz, fixations and saccadic movements are derived from that. Stimuli images were presented on a 4 K 27” (3840 × 2160, 163 PPI, 60 Hz refresh rate) IPS screen with 100% sRGB color space. The screen was color- and luminance-calibrated with X-Rite i1 Display Pro probes connected during the whole rating session to adjust the screen for ambient light. The eye tracking and rating session took place in a quiet and windowless eye tracking lab in standardized conditions across all raters. Raters were seated with their heads on a chin and forehead rest (SR Research Head Support) 70 cm from the screen. Every participant saw images in a randomized order, with instructions to freely observe image on the computer screen.

We determined the dominant eye of each participant using a variation of the Porta test^115^. Although vision is binocular, we tracked only the dominant eye. The eye tracker was calibrated by a standard nine-point routine. Calibrations was validated by the EyeLink software and repeated as necessary until the optimal calibration criterion is reached.

Each image begun with a drift correction. A fixation cross on an 18% grey background appeared (in eight possible positions) on the screen, and participants were instructed to focus their gaze on it. The distance of the centers of the corner crosses from the center of image was 1275 pix at angles of 155°; −155°; 25°; −25°. The centers of the crosses above and below the image center were 542 pix at angles of 90° and −90°. The centers of the crosses to the right and left of the image center were 1150 pix at the angles of 0° and 180°. The cross size was 183 pix with a stroke thickness of 7 pix. The semi-random position of the cross out of the center was chosen to avoid visual bias towards the center of the image. When a participant’s eye fixates on the cross, the stimuli presentation will initiate for five seconds.

### Symptom rating and cognitive testing

After conducting the eye-tracking measurements, we utilized the Positive and Negative Syndrome Scale (PANSS)^116^ to assess the severity of positive and negative symptoms in SCHZ patients. Additionally, we employed Conners’ Continuous Performance Test III (CPT)^117^ to evaluate attention. We hypothesized that diminished attention, as indicated by the CPT, would influence perception processing, given that visual attention is crucial for acquiring information visually^117^. These assessments were conducted at the National Institute of Mental Health (NIMH CZ) in a quiet, dedicated room. The entire assessment process, led by a trained psychologist, lasted approximately 2 hours. The primary objective of this psychological testing was to investigate any potential causal links between the illness, the performance of the saliency models, and the oculomotor behavior observed in the patients.

### Data pre-processing and statistics

Primary pre-processing (differentiation between saccades and fixations) was performed in the EyeLink Data Viewer. The data were then exported to a spreadsheet format (CSV) for further processing. In the first step, all ET data were cleaned of off-monitor fixations and saccades. The first fixation overlapping with the fixation cross between stimuli was removed and no longer considered. Pre-processing and all table data (including PANSS, CPT, saliency prediction scores, and demographic data) were statistically analyzed with R^118^ using the tidyverse package^119^.

**Fig. 4:**
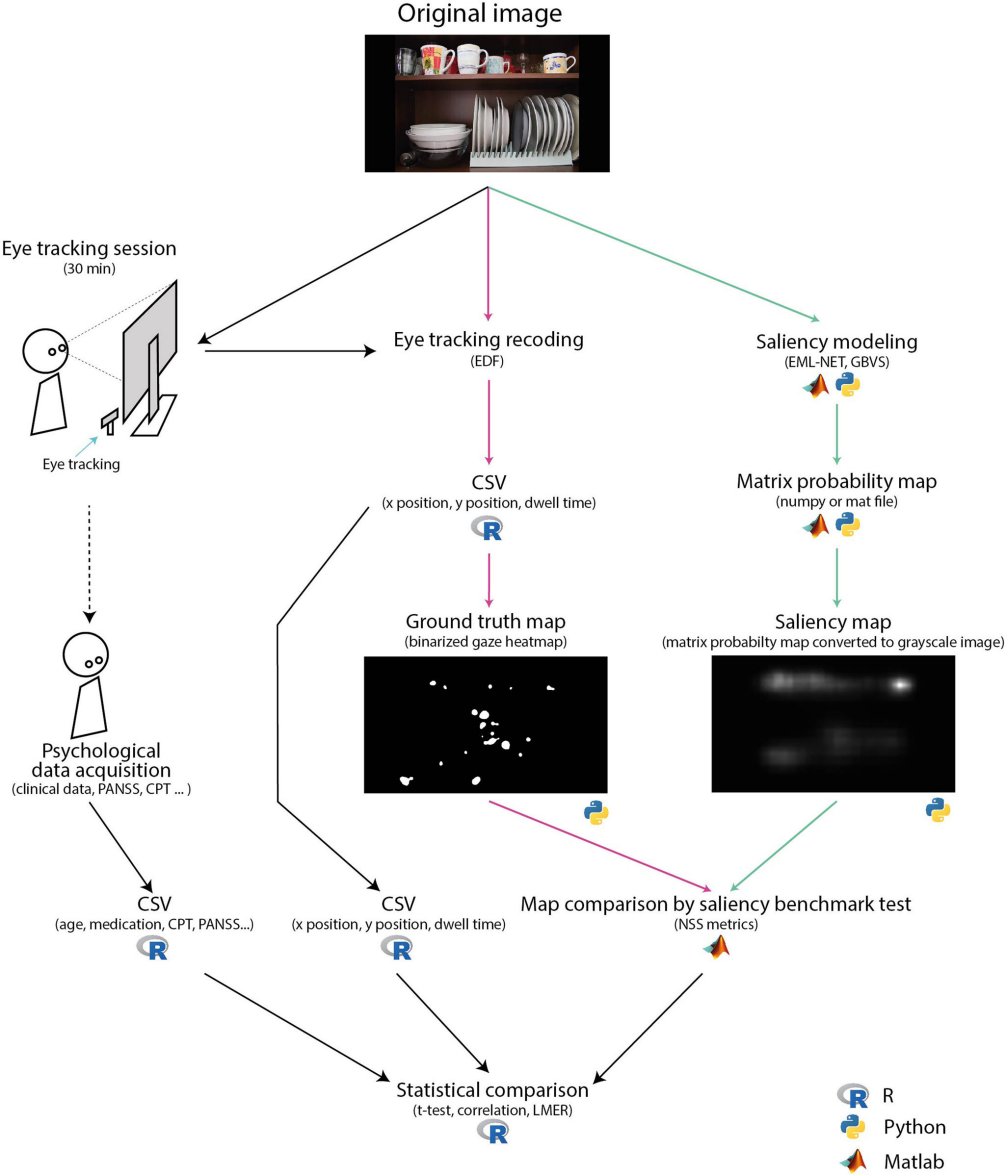
The diagram illustrating data processing and analysis steps utilized in the study. Pink arrows mark the processing path of the ground truth map. Green arrows mark the processing path of the saliency models. Black arrows mark the processing path of table data for statistical comparison; CSV comma-separated values, EDF standardized European data format for storage of medical time series, NSS normalized scan path saliency, PANSS Positive and Negative Syndrome Scale, CPT Conners’ Continuous Performance Test III.

Ground truth fixation matrices were calculated from the cleaned fixation data for each participant and image in Python using the GazePointHeatMap package^120^. This matrix contains the fixation averages for each image area over time. Ground truth fixation map was in full resolution of the original stimuli (3840 × 2160). Two subsequent ground truth maps from fixations were computed (up to the fifth fixation and from the sixth fixation) to examine whether the bottom-up signal bias in the SCHZ group persists over time or not. Python was used to process both saliency models, which are published at github.com (GBVS^121^; EML-Net^122^). The final performance evaluation of each saliency model was calculated using the MIT saliency benchmark toolbox^40^ in MATLAB (Fig. 4).

The inter-group difference in the total examined image area was calculated using the standard distance deviation formula (SDD) in R with the mapTool package^123^. We investigated the relationship between the oculomotor behavior of SCHZ patients and key clinical factors: the duration of untreated psychosis and the chlorpromazine equivalent^54,124,125^ were investigated in R.

Finally, the metrics differences between-groups were evaluated using Linear Mixed-Effects Models (R lme4 package)^126^. The models used NSS metrics value as the dependent variable and included fixed effects for interaction between-group (patients vs controls), image category, crossed random intercepts for each individual (participants ID) and each image category. Estimating random intercepts for individual images was not feasible due to the extensive number of parameters required. Prior to modelling, the NSS score was transformed using square root transformation to suppress skewness of the distribution. Inputs and resulting distributions, as well as model residuals, were checked using density and q-q plots. Significance tests on fixed effects were performed using Satterthwaite’s method (R lmerTest package)^127^.

The Wilcoxon signed-rank test was applied to assess saccadic eye movement, which had a non-normal distribution. A Pearson’s correlation test was used to assess the association between medication, the outcomes of psychological tests, and the duration of untreated psychosis with the findings of the oculomotor movements. For all the tests, the significance level was set at alpha < 0.001 in order to take into consideration multiple comparisons.

For the between-group comparison of fixation duration, we used the sequential testing procedure: starting from fixation 1, the between-group differences were compared using the t-test at a significance level alpha = 0.05. The subsequent fixations were considered significant if, and only if, current and all preceding tests rejected the null hypothesis. This approach conforms to the closed testing procedure and thus controls the overall significance level at alpha = 0.05^128^.

### Saliency Models

The selection of the most recent top-down and bottom-up saliency models used in our study was based on the models’ overall success in their category as measured by the MIT Saliency Benchmark (saliency.mit.edu)^40^. We selected the best-performing models from the top-down and bottom-up categories based on the NSS metrics^129–132^, which was set as a mandatory performance indicator at the 14^th^ European Conference on Computer Vision^40^. The second criterion was the availability of source code. We chose the results from a MIT300 dataset^131^, which by its nature, better reflects the stimuli used in our study than a CAT2000^133^, which contains only natural scenery.

As the bottom-up model, we selected the pre-trained GBVS^134^, which works by constructing a graph representation of the image, where each node in the graph corresponds to a small region of the image. This process consists of two steps. First, it creates numerical activation maps of feature channels extracted from locations in the image (e.g., by linear filtering followed by elementary nonlinear filtering). Second, it normalizes the activation maps in a way that emphasizes conspicuity and allows combinations with other maps^135^. The model takes a Markovian approach at both steps. Markov chains are defined over various image maps, and the equilibrium distribution over map locations is treated as activation and saliency values. The edges between the nodes represent the similarity between the regions. The model then computes a saliency value for each node based on its contrast with neighboring regions. The nodes with high saliency values are considered to be the most visually salient regions of the image and are likely to attract human attention.

As the top-down model, we selected the pre-trained EML-Net^136^, a deep-learning model used for image saliency prediction. The EML-Net model uses CNN layers to extract features from the image and then passes these features through multiple layers of fully connected neural network layers to predict the saliency. Specifically, the encoder consists of NasNet from ImageNet and DenseNet from PLACE365^136^, both are used as encoder for image classification. During training, the model learns to predict the saliency map for a given input image by adjusting the weights of the neurons in the network to minimize the difference between the predicted saliency map and the ground truth map.

To enable a meaningful comparison between two distinct prediction models, the NSS metrics were selected to evaluate their performance^40^. Specifically, NSS metrics measure accuracy by comparing the predicted saliency map created by the model with the fixation density map from eye-tracking data (ground truth map).^129^ The fixation density map shows where viewers look at an image. NSS calculates the mean saliency value at the fixated locations by comparing the predicted map with a binary fixation map, where ‘ones’ represent fixations and ‘zeros’ represent other areas^137^. A higher NSS value suggests a better prediction of viewer attention, while a value of zero indicates chance-level predictions. NSS is widely used for comparing different saliency models because it provides a straightforward and standardized way to assess their performance.

### Supplementary information

Apendix A

## Data Availability

The data analyzed during the current study are available from the corresponding author upon reasonable request. Analysis scripts are available on the OSF: https://osf.io/hz2p8/.
