# Supplementary material for: The Gaze of Schizophrenia Patients Captured by Bottom-up Saliency
Source: Schizophrenia (Heidelb). 2024 Feb 20;10(1):21. doi: 10.1038/s41537-024-00438-4 (PMC10879495; doi:10.1038/s41537-024-00438-4)
Supplement: Supplementary file 1 — Apendix A [file 41537_2024_438_MOESM1_ESM.docx]

**Appendix A**

Power analysis:

The sample size was estimated at 18 subjects per group based on the results of a pilot study (n = 8), previous literature (1, 2) and power analysis. The level of confidence was set at 80%, and the statistical significance level was set at p = 0.05. Considering the expected dropout rate of approximately 10% and potential problems with the quality of the data, we planned to measure 20 subjects for each group. The number of patients was intentionally overestimated because we expected a higher variability within this group and a higher dropout rate.

1. Sprenger A, Friedrich M, Nagel M, Schmidt CS, Moritz S, Lencer R (2013): Advanced analysis of free visual exploration patterns in schizophrenia. *Frontiers in psychology*. 4:737.

2. Beedie SA, Benson PJ, Giegling I, Rujescu D, St. Clair DM (2012): Smooth pursuit and visual scanpaths: independence of two candidate oculomotor risk markers for schizophrenia. *The world journal of biological psychiatry*. 13:200-210.
